# Recent Progress toward Microfluidic Quality Control Testing of Radiopharmaceuticals

**DOI:** 10.3390/mi8110337

**Published:** 2017-11-21

**Authors:** Noel S. Ha, Saman Sadeghi, R. Michael van Dam

**Affiliations:** 1 Department of Bioengineering, Henry Samueli School of Engineering and Applied Science, University of California Los Angeles, Los Angeles, CA 90095, USA; noelha@ucla.edu; 2 Crump Institute for Molecular Imaging and Department of Molecular and Medical Pharmacology, David Geffen School of Medicine, University of California Los Angeles, Los Angeles, CA 90095, USA; samsadeghi@mednet.ucla.edu

**Keywords:** radiopharmaceuticals, pharmaceuticals, microfluidics, quality control (QC) testing, positron emission tomography (PET), single photon emission computed tomography (SPECT), lab-on-a-chip, sensor

## Abstract

Radiopharmaceuticals labeled with short-lived positron-emitting or gamma-emitting isotopes are injected into patients just prior to performing positron emission tomography (PET) or single photon emission tomography (SPECT) scans, respectively. These imaging modalities are widely used in clinical care, as well as in the development and evaluation of new therapies in clinical research. Prior to injection, these radiopharmaceuticals (tracers) must undergo quality control (QC) testing to ensure product purity, identity, and safety for human use. Quality tests can be broadly categorized as (i) pharmaceutical tests, needed to ensure molecular identity, physiological compatibility and that no microbiological, pyrogenic, chemical, or particulate contamination is present in the final preparation; and (ii) radioactive tests, needed to ensure proper dosing and that there are no radiochemical and radionuclidic impurities that could interfere with the biodistribution or imaging. Performing the required QC tests is cumbersome and time-consuming, and requires an array of expensive analytical chemistry equipment and significant dedicated lab space. Calibrations, day of use tests, and documentation create an additional burden. Furthermore, in contrast to ordinary pharmaceuticals, each batch of short-lived radiopharmaceuticals must be manufactured and tested within a short period of time to avoid significant losses due to radioactive decay. To meet these challenges, several efforts are underway to develop integrated QC testing instruments that automatically perform and document all of the required tests. More recently, microfluidic quality control systems have been gaining increasing attention due to vastly reduced sample and reagent consumption, shorter analysis times, higher detection sensitivity, increased multiplexing, and reduced instrumentation size. In this review, we describe each of the required QC tests and conventional testing methods, followed by a discussion of efforts to directly miniaturize the test or examples in the literature that could be implemented for miniaturized QC testing.

## 1. Introduction

**Figure 1:**
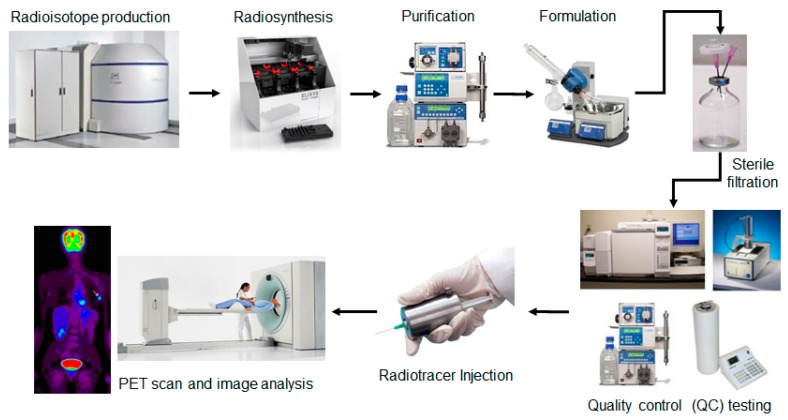
Production of positron emission tomography (PET)/single photon emission tomography (SPECT) radiotracers for clinical imaging involves generation of the radioisotope (via cyclotron or generator), radiosynthesis, purification (via HPLC or solid-phase extraction), formulation (via evaporative or solid-phase extraction methods), followed by quality control (QC) testing to ensure safety of the formulated radiotracer prior to injection.

Positron emission tomography (PET) and single photon emission tomography (SPECT) are real-time, 3D imaging techniques that have unparalleled specificity and sensitivity for visualizing biochemical processes in living subjects [1,2]. The information from a PET or SPECT scan is used clinically in disease diagnosis, prediction of response to therapy, and monitoring of response to therapy [1,3,4,5,6,7]. PET is also an indispensable research tool for uncovering mechanisms of disease initiation and progression, developing new therapies, measuring and optimizing the pharmacokinetic properties of new therapeutic compounds, and evaluating new therapies in clinical trials [8]. PET and SPECT both rely on the injection of a radioisotope-labelled compound, known as a radiotracer or radiopharmaceutical, into the patient which targets specific receptors, enzymes, or proteins and allows their position and density/activity to be measured via radiation detectors during a PET or SPECT scan. PET employs radiotracers labelled with positron (β+)-emitting isotopes (e.g., F-18, C-11, O-15, N-13, Ga-68, Cu-64, Zr-89, etc.), which release positrons upon decay. These positrons rapidly annihilate with nearby electrons to form two anti-parallel gamma (γ) rays (511 keV each) that are detected by a ring of detectors [2]. SPECT typically utilizes radioisotopes that directly emit γ rays (e.g., Tc-99m, I-123, In-111, etc.) which are detected using a collimator and gamma camera [9]. In both cases, many decay events are detected to enable reconstruction of the three-dimensional radiotracer distribution in the body. Due to the short half-life of PET and SPECT radioisotopes, labeled tracers must be manufactured just prior to imaging (Figure 1). And because these compounds are injected into humans, there are strict regulatory requirements for performing quality control (QC) testing of the purified, formulated product of each batch that is produced, to ensure safety before they can be released for clinical use [10,11]. Specific procedures and requirements for these tests are described in regulatory documents published in each country or region (e.g., U.S. Pharmacopeia (USP) General Chapter <823> [12] and U.S. Food and Drug Administration (FDA) 21 CFR Part 212 [13]; European Pharmacopoeia (EP) [14], etc.). Procedures specific to the PET tracer 2-[^18^F]fluoro-2-deoxy-D-glucose ([^18^F]FDG) have been discussed in detail in several review articles [15,16]. In general, pharmaceutical tests (e.g., pH, color and clarity, chemical purity, residual solvents, pyrogenicity, sterility) are required to ensure physiological compatibility of the final preparation and the absence of microbiological, pyrogenic, chemical or particulate contamination. In addition, radioactive tests (e.g., radiochemical identity, radiochemical purity, radioisotope identity, radioisotope purity, and radioactivity concentration) are required to ensure there are no radioactive or radionuclidic impurities that could interfere with the biodistribution or imaging protocol and to ensure the proper patient dose [17].

Performing and documenting these required QC tests is cumbersome and time-consuming, and requires an array of expensive analytical chemistry equipment and significant dedicated lab space. In addition, for most tests, manual handling, loading, and/or visual assessment of samples leads to significant radiation exposure to personnel and operator-induced variability in the results. In particular Ferguson et al. found that QC personnel received significant radiation exposure, especially due to performing sterility (filter integrity), pH, and chemical/radiochemical purity and identity testing [18]. Furthermore, in contrast to ordinary pharmaceuticals, each batch of short-lived PET radiopharmaceuticals has to be manufactured and tested within a short period of time to prevent significant losses due to radioactive decay [17].

Several companies, including QC-1 (Munster, Germany) [19], Trace-ability (Culver City, CA, USA) [20], and ABT Molecular Imaging Inc. (Louisville, TN, USA) [21,22], have been developing QC systems that automatically perform the tests and also handle the majority of the needed equipment calibrations, performance testing, and report generation. After further development and appropriate validation, such automated QC testing systems have the potential to significantly alleviate the labor burden and decrease radiation exposure.

More recently, there have been efforts to miniaturize the instrumentation for QC testing by using microfluidics, much like the efforts to miniaturize other stages of radiotracer production (radioisotope concentration, radiosynthesis, purification, and formulation) [23,24,25,26,27]. Microfluidic QC systems could lead to significantly lower instrumentation cost and reduction of needed laboratory space. Microfluidic techniques in general also offer many other important advantages over their conventional counterparts, including vastly reduced sample and reagent consumption, shorter analysis times, higher detection sensitivity, and increased multiplexing or parallelism [26,28]. Furthermore, the fabrication and material cost of many techniques used in microfluidic QC systems can be very low, potentially enabling tests to be implemented with a disposable fluid path. These advantages could be especially helpful in conjunction with emerging technologies that produce smaller batches of PET/SPECT tracers at a time (each requiring QC testing), including dose-on-demand approaches [29].

While the field is still far from achieving a fully-integrated microscale QC testing platform, we highlight in this review the significant progress that has been made in developing microscale implementations suitable for several of the required QC tests for radiopharmaceuticals and pharmaceuticals.

## 2. Miniaturization of Quality Control Tests

Testing of PET and SPECT radiopharmaceuticals involves several specific measurements to ensure product purity, identity, and biological safety for human subjects [12,14]. The detailed criteria for passing each test depend on the particular radiopharmaceutical and method of preparation, but in many cases the tests are similar or identical across a broad range of radiotracers. Below, we describe each of the required tests and the conventional method(s) of performance, and then describe current efforts to directly miniaturize each test or discuss examples from the field of microfluidics that could be implemented to miniaturize the test.

### 2.1. pH Test

pH must be controlled to ensure both the stability of the formulated radiopharmaceutical (to ensure it does not degrade between manufacturing and injection) and its physiological compatibility with the patient. Due to the high buffer capacity of the blood and the relatively small injection volume (typically 1–10 mL), a relatively wide pH range (typically 4.5–8.5) is considered to be acceptable from a physiological point of view [17]. The requirement for stability often shrinks the acceptable range. The pH of the formulated radiotracer is most commonly measured using a calibrated electronic pH meter or pH indicator strips.

**Figure 2:**
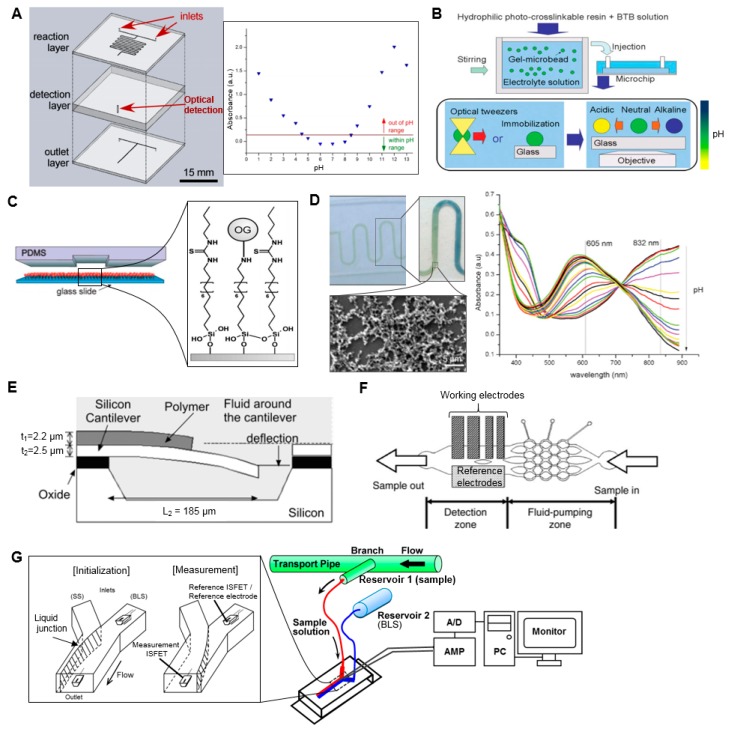
Examples of microfluidic pH measurement systems. (**A**) (**Left**) Microfluidic device to mix a sample with a universal pH indicator and then measure optical absorbance in a 3.1 mm long detection cell. (**Right**) Representative absorbance measurements at 551 nm as a function of sample pH. Adapted with permission from [31] Copyright © 2014 The Chemical and Biological Microsystems Society. (**B**) (**Top**) pH-sensing polymer microbeads are prepared by introducing the pH indicator during the cross-linking process. (**Bottom**) The beads are immobilized using optical tweezers or tethered to the surface, and the color of each bead reflects the pH of the local environment. Adapted from [34] with permission of The Royal Society of Chemistry. (**C**) Microfluidic device with internal channel surface coated with pH-sensitive fluorescent molecules (Oregon Green 514). The fluorescence intensity is correlated with pH. Adapted from [36] with permission of The Royal Society of Chemistry. (**D**) (**Left**) polydimethylsiloxane (PDMS) microfluidic channel with polyaniline coating that changes color in response to pH. The zoomed in image shows the response to a pH gradient along the channel. Inset SEM image shows the structure of the coating layer. (**Right**) Absorbance spectra of the polyaniline coating when exposed to solutions of different pH. Adapted from [37] with permission of The Royal Society of Chemistry. (**E**) Micro-cantilever undergoes deflection in response to pH-dependent swelling of a hydrogel polymer coating. Deflection is monitored with a laser beam. Adapted with permission from [40] Copyright © 2003 Springer. (**F**) Thin-film electrodes integrated into a PDMS chip allow pH to be measured based on potential between working and reference electrodes. Adapted with permission from [41] Copyright © 2006 Elsevier. (**G**) Flow-based microfluidic pH measurement system using ion-sensitive field-effect transistor (ISFET) sensors, one acting as a reference and one as a working electrode. Modulation of flow rates moves the liquid junction and allows measurement of the sample solution (SS) after an initialization process using a baseline solution (BLS). Adapted from [42] with permission from MDPI AG.

While there are a myriad of approaches and technologies developed for the measurement of pH, to the best of our knowledge, there is only one report (aside from a brief mention in a patent [30]) on application of a microscale approach to pH measurement of radiopharmaceuticals. Tarn et al. described a simple, 3-layer, glass microfluidic device, in which the sample is first combined with a universal pH indicator in an on-chip mixer, and the optical absorbance of the mixture is measured with a spectrometer connected via optical fibers to an extended flow-cell in the chip (Figure 2A) [31]. The absorption spectrum of the indicator shifts as a function of pH, and a calibration was created using reference standards to relate the pH value to the absorbance at that wavelength. With this device, the pH of small samples (<2 µL) of [^18^F]FDG solutions could be determined (in the pH range 1–13) within a few minutes. A second analysis method was also reported, where the absorbance was monitored at multiple wavelengths to obtain a “fingerprint” of the sample, which could be “matched” with fingerprints of standards. The flow-cell is an important part of the implementation as the small dimensions of microchannels can lead to low optical path lengths (OPL) and thus low absorbance values and low detection sensitivity. A variety of detection cell designs have been reported in the literature that incorporate (i) an extended optical path to increase absorbance by the sample; (ii) multiple passes of the illumination light through the sample to increase absorbance; or (iii) an optical resonator that is filled with the sample. These approaches were reviewed by Rushworth et al. [32].

A wide variety of other microfluidic methods for measuring sample pH have been reported [33]. These include miniaturized approaches based on traditional pH indicators or electrochemical cells, as well as approaches based on detection of physical or optical changes in pH-responsive coatings. Many of these approaches could presumably be applied to pH analysis of radiopharmaceuticals, provided they have a sufficiently wide working pH range and fast analysis time. A few examples of these approaches are discussed below.

Maruyama et al. used gel microbeads impregnated with pH indicator to measure local pH in the sample immediately surrounding the bead (Figure 2B) [34]. The gel microbeads, positioned with optical tweezers, changed color within a few seconds when in contact with sample solution indicating the local pH over the range 5.8–9.0. Maclin et al. reported a similar approach in which the indicator was contained in nanocapsules immobilized in high-porosity polyvinyl alcohol matrix [35], and demonstrated analysis of 30 µL sample volumes within 2 min spanning a pH range of 2.7 to 12.6. An advantage of these approaches is more accurate measurements (since they avoid adding the indicator into solution, which, in high quantities, can affect the pH being measured). Furthermore, because mixing is not required, the implementation can be simpler.

Other approaches for pH measurement are based on detectable changes in a pH-sensitive coating. For example, Mela et al. reported the detection of pH by modifying the internal surface of a microfluidic channel with the pH-sensitive fluorescent dye Oregon Green 514 (Figure 2C) [36]. By monitoring fluorescent emission intensity, pH in the range 2–10 could be measured in real time as an aqueous sample flowed through a 20 µm × 2 µm channel inside a polydimethylsiloxane (PDMS)/glass chip. Florea et al. described a device in which the surface of a microchannel was coated with polyaniline [37]. pH of the sample flowing through the device affects the optical properties of the polymer coating resulting from a reversible protonation/deprotonation reaction. Response could be detected over the pH range 2–12 in real time (Figure 2D).

In addition to optical changes, pH can induce changes in density, volume, and stiffness of coatings such as hydrogels that can be detected as changes in refractive index, electrical resistance, natural frequency of mechanical oscillation, etc. These approaches have been described in a comprehensive review [38]. In one example of these approaches, Trinh et al. reported a hydrogel-based piezoresistive pH sensor, in which a hydrogel was placed between a stiff, porous grate and a piezoresistive bending plate transducer [39]. pH-induced swelling of the hydrogel deflected the plate and caused a detectable resistance change, allowing detection of pH values in the range 5.5–11 within 12 min. In a similar approach, Hilt et al. [40] reported the use of a hydrogel coating on a micro-cantilever that could respond to changes in environmental pH resulting in a surface stress that deflected the cantilever (Figure 2E). By measuring the deflection with a laser beam reflecting from the cantilever surface, pH measurements in the range 2.8–6.8 were reported.

pH can also be detected via electrochemical reactions, in which the electrical potential is sensitive to the pH of the sample solution in the electrochemical cell. A wide variety of implementations of electrochemical cells have been reviewed [33,40] and a few examples of microscale cells reported for pH measurement are given below. Lin et al. developed a microfluidic continuous-flow pH measurement chip by integrating thin-film pH-sensing electrochemical electrodes into in a PDMS chip (Figure 2F) [41]. The free hydrogen atoms of the sample react with the sensing electrodes (fabricated by sputtering layers of SiO_2_–Li O_2_–BaO–Ti O_2_–La_2_O_3_ (SLBTLO) on platinum (Pt) electrodes) inducing a detectable change in potential with respect to the reference electrode (Ag electrode with thin coating of AgCl). pH measurements in the range pH 2 to 10 could be made using only ~0.5 µL sample volume and 200 s duration. Yamada and Suzuki developed a flow-based microfluidic pH measurement system using ion-sensitive field-effect transistor (ISFET) sensors for measurement of the proton concentration (Figure 2G) [42]. Two ISFET sensors and an Ag/AgCl pseudo reference electrode are fitted into a microfluidic Y-junction such that the reference ISFET and reference electrode are always immersed in a stream of baseline solution and the measurement ISFET is immersed in baseline solution or the sample, depending on a switchable flow. Under a continuous flow, sample solution could be measured in <120 s with a wide detection range (pH 1.68–10.0). Though the authors reported 2.0 mL sample consumption per measurement due to filling and flushing the external tubing and syringe pumps, the dimensions of the microfluidic chip suggest the detection volume was <14 µL, a value that can be compared with other papers.

### 2.2. Appearance Test (Optical Clarity Test)

In general, only a clear and colorless, particulate-free solution should be used for injection. Formulated PET and SPECT tracers are generally colorless due to the lack of appreciable absorbance by the tiny amounts of tracer used (e.g., pmol to nmol for a ~370 MBq (10 mCi) single patient dose of a ^18^F-labeled PET tracer, or nmol to µmol for a >37 GBq (>1 Ci) multi-dose batch). Any coloration would indicate a significant quantity of an impurity. Generally, the test is performed manually, via qualitative visual inspection, resulting in variability in the readout.

Using the same microfluidic device as described above for pH testing, Tarn et al. reported automation of a quantitative optical clarity test for [^18^F]FDG [31]. A non-clear sample could be detected when the absorbance exceeded that of a reference solution (water). Though this appears to be the only microfluidic implementation of the appearance test applied specifically to the analysis of radiopharmaceuticals, it is likely that any of the other strategies described above based on optical absorbance could also be used to implement a miniature optical clarity test based on the same principle.

### 2.3. Sterility Test

According to the FDA document “current good manufacturing practice for PET drugs” [43], even if care is taken to minimize microbiological contamination during synthesis, a drug is considered to be nonsterile until it is passed through a sterilizing grade filter. Generally, radiopharmaceutical production can use commercially available, pre-sterilized filters, provided that the vendor has been shown to be reliable and the filter meets certain specifications. Conventionally, sterility is assessed by inoculating the filtered sample into two types of culture media (soybean-casein digest medium (SCDM) to culture aerobic bacteria and fungi, and fluid thioglycollate medium (FTM) to culture anaerobic bacteria), incubating for ≥14 days, and then looking for formation of colonies [44]. However, because this timeframe is much longer than the half-life of SPECT and PET radioisotopes, the FDA allows a quick, short-term test to be used to enable early release of the radiotracer (though the culture test must still be completed). In the short-term test, the integrity of the filter membrane is assessed (typically via a bubble point test) after completing sterile filtration. In this test, compressed gas is applied to the inlet of the wetted filter and pressure is increased until bubbling appears at the outlet (i.e., the bubble point). If the bubble point exceeds a threshold pressure, then it can be assured that the membrane is intact and pores do not exceed the specified size. A drawback of this test is that the operator has to manually handle the filter membrane and it has been reported that this test results in the largest radiation dose to QC personnel [18].

Although not yet demonstrated specifically for the analysis of radiopharmaceuticals, microfluidics may provide a way to directly assess sterility in a much shorter time than 14 days, and potentially obviate the need for the bubble point test. In the conventional culture test, the long duration is essentially needed for amplification, i.e., to allow many cycles of growth, such that any bacterial colonies could be detected visually. This growth amplification step could be omitted for high-sensitivity microfluidic approaches that enable direct detection of individual microorganisms in liquids [45].

A large number of reports (reviewed in [45,46,47,48]) have demonstrated the detection of small numbers of bacteria (even single cells). Some strategies rely on PCR amplification of specific DNA sequences, or sensitive assays of specific surface antigens or metabolites. In one example, Jung et al. reported an integrated microfluidic device capable of detection and identification of as few as 1 colony-forming unit (CFU) in a 10 µL sample via a sandwich-type assay within 30 min [49]. In the assay, bacteria are bound by two types of particles functionalized with antibodies that target the bacteria. The magnetic particles allow the bacteria to be immobilized using a magnetic field while unbound particles were washed away, and then “barcode” DNA was released from the other particle for analysis by an electrophoretic separation and detection unit. Despite impressive sensitivity and operation speed, such methods may not be suitable for QC testing of radiopharmaceuticals because they detect only the specifically-targeted pathogens rather than all pathogens.

**Figure 3:**
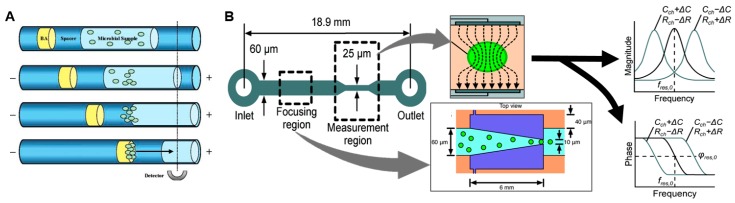
Microfluidic methods for detection of bacteria. (**A**) A microbial sample with “universal” fluorescent stain is concentrated by capillary electrophoresis to improve detection sensitivity. (BA = “blocking agent”). Adapted with permission from [50] Copyright © 2007 American Chemical Society. (**B**) Impedance-based bacterial detection chip. Cells are first focused via dielectrophoresis (DEP) to the center of channel for downstream detection. A detectable shift in resonant frequency and phase occur when a single cell or particle passes through the measurement area due to a change in the channel impedance. Adapted from [53] with permission of The Royal Society of Chemistry.

This can be partially addressed by incorporating certain types of “universal” PCR primers or bacterial stains that target broad classes of pathogens. For example, Lantz et al. reported a high-sensitivity microfluidic device capable of detecting diverse bacteria and fungi down to the single cell level [50]. The sample was stained with the dye BacLight Green, then a series of plugs (sample, buffer, and “blocking agent”) were injected into a capillary/channel causing aggregation of cells when electrophoretic potential was applied (Figure 3A). The concentration process, combined with fluorescence measurement over a 10 min integration time resulted in high detection sensitivity.

Rather than relying on specific biochemical markers, other rapid approaches for bacterial detection have been reported that are based on detection of physical properties such as electrical impedance of single cells (reviewed in [51,52]). In impedance-based flow cytometry, the sample solution is focused down into a narrow channel containing a detector such that at most only one cell at a time flows over a region with detection electrodes. When there is a cell within the detection region, the impedance differs from that of the buffer due to capacitance of the cell membrane and possibly differing resistance of the cell contents. Haandbæk et al. reported a resonance-enhanced microfluidic impedance cytometer for detection of single bacteria (Figure 3B) [53]. After focusing cells to the center of the channel via dielectrophoresis (DEP), bacterial cells passing over downstream electrodes were detected and characterized by frequency and phase shifts an electrical resonator. With the sample flowing at a speed of 0.5 µL/min, the system was able to detect single particles as small as 0.9 µm in diameter, as well as distinguishing different sizes and types (bacteria or polymer bead) of particles.

Though extensive validation and other work would be needed, it seems that microfluidic approaches like impedance cytometry might be capable of performing a rapid sterility test by counting the number of bacterial cells in a sample if throughput can be increased to measure a sufficiently large representative sample (e.g., 100 µL) of the radiopharmaceutical formulation without compromising detection sensitivity. If such a technique could be realized, there would potentially be no need for the filter integrity test.

### 2.4. Bacterial Endotoxin Test

Bacterial endotoxins, lipopolysaccharides (LPS), are toxic components of the outer membrane of gram-negative bacteria that can cause fever and possibly leukopenia in immunosuppressed patients [17]. Because endotoxins can contaminate a solution even if the bacteria have been thoroughly removed via sterile filtration, it is necessary to test radiopharmaceutical formulations for their presence. According to the USP Bacterial Endotoxins Test (General Chapter <85>) [54], the maximum allowable endotoxin level in radiopharmaceutical injections is 175 EU/V, where V is the maximum volume (mL) of drug administered at the time of expiration (1 EU = 100 pg of Escherichia coli LPS). The conventional test for bacterial endotoxins is based on a multi-step biochemical pathway leading to activation of a clotting enzyme that occurs when bacterial endotoxins are mixed with Limulus amebocyte lysate (LAL) derived from blood cells of horseshoe crabs (Limulus polyphemus or Tachypleus tridentatus) [55]. The enzyme acts on a coagulogen to create a “clot” in the test sample (“gel clot method”) or to increase its turbidity which can be optically detected (“turbidimetric method”). Alternatively, if a chromogenic substrate is added, a color change can be detected (“chromogenic method”). The timescale of clotting, turbidity increase, or color change is related to the endotoxin concentration. Several commercial reagent kits and test instruments are currently available and are routinely used in QC testing of radiopharmaceuticals [55].

**Figure 4:**
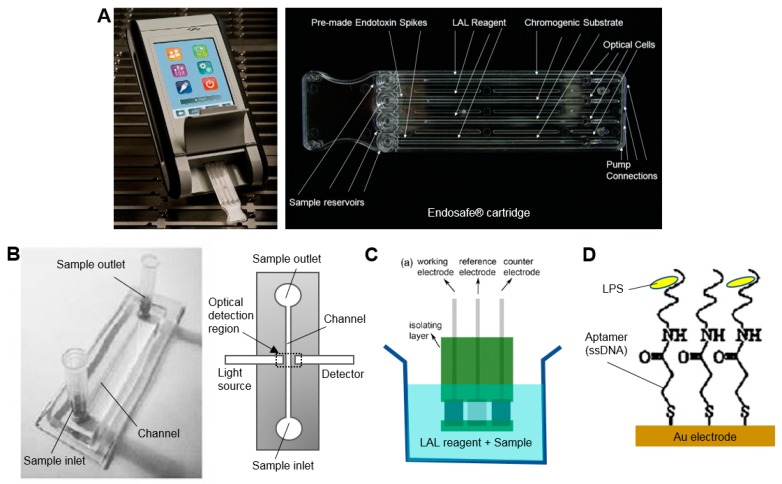
Examples of microfluidic devices for bacterial endotoxin test. (**A**) Commercial LAL assay system (Endosafe^®^, Charles River Laboratories, Inc.) comprising a portable, hand-held spectrometer (**left**) and ~25 mm × 75 mm disposable microfluidic cartridge (**right**). Adapted from [56]. (**B**) Photograph of microfluidic LAL test chip (**left**) and block diagram illustrating detection method (**right**). Sample mixed with chromogenic substrate is loaded into the microchannel and optical detection is performed in the middle of channel. Photograph adapted with permission from [57] Copyright © 2004 Springer. (**C**) Screen-printed electrodes for electrical-impedance monitoring of standard gel clot LAL assay. Adapted with permission from [58] Copyright © 2013 Elsevier. (**D**) Biosensor comprising a gold electrode functionalized with endotoxin-specific aptamer. Binding of endotoxin is detected via impedance spectroscopy. Adapted with permission from [60] Copyright © 2012 Elsevier.

One of these commercial systems (EndoSafe, Charles River Laboratories, Wilmington, MA, USA), already in widespread use in testing of radiopharmaceutical formulations, performs a small-scale version of the chromogenic assay in 15 min using disposable ~25 mm × 75 mm microfluidic cartridges pre-loaded with reagents (Figure 4A) [56]. In this system, a 25 µL sample is loaded on the cartridge, mixed with the LAL reagent and then combined with the chromogenic substrate, incubated, and finally the color intensity is measured over time via a small handheld spectrophotometer-based reader. This system can detect down to 0.005–10 EU/mL. Though not specifically applied to radiopharmaceutical analysis, one group demonstrated a similar assay in a microfluidic chip (18 mm × 62 mm) made of PDMS (Figure 4B) [57]. After mixing the sample with reagents outside the chip, a 4 µL sample was injected and result could be obtained after 10 min. To match the capabilities of the EndoSafe device, the PDMS chip would have to be modified to include analysis of a replicate as well as positive controls.

Miao et al. reported an electrochemical approach that replaced the optical readout of the standard gel clot assay with an electrical readout [58]. Millimeter-sized screen-printed electrodes were inserted in a small volume of a mixture of the sample and LAL reagent (270 µL) at 37 °C and the electrical current was monitored over time (Figure 4C). Onset of gel clotting resulted in a rapid drop in current, the timing of which could be correlated with endoxtoxin concentration. Response time was fast (<100 s) and a detection limit of 0.03 EU/mL was reported.

In addition to miniaturized versions of the standard LAL assay, there has been considerable development of new approaches, including modifications to the standard assay to improve sensitivity, as well as miniature biosensors that detect endotoxins based on binding with surface-immobilized biomolecules (recently reviewed in [55]).

For example, Noda et al. a reported a modified LAL assay in which the coagulogen was replaced with a luciferin-modified peptide (benzoyl-Leu-Gly-Arg-aminoluciferin) [59]. When this substrate becomes activated by the LAL cascade (i.e., in the presence of endotoxin), the reaction with luciferase produces luminescence, allowing very sensitive detection. A detection limit of 0.0005 EU/mL was reported in an assay time of 15 min. Though such high sensitivity is not strictly necessary, it may provide the capability to use a smaller sample volume.

Sensor-based methods rely on detection of endotoxin binding, and can produce a signal via fluorescence or luminescence assays, electrical impedance, electrochemical reactions, or mechanical resonators. Though many of the approaches require lengthy procedures to modify/derivatize the LPS prior to detection or have poor sensitivity, there are a few approaches that appear sufficiently fast and sensitive for QC testing of radiopharmacuticals. For example, Su et al. demonstrated an impedance-based readout with a detection limit of 0.05 EU/mL within 10 min [60]. The electrochemical biosensor comprised a gold electrode functionalized with an LPS-specific single stranded DNA (ssDNA) aptamer as a probe (Figure 4D). A good linear relationship between charge-transfer resistance and logarithm of LPS concentration was demonstrated over a wide dynamic range (0.01–10 EU/mL). As surface binding sensors are often prone to fouling, non-specific binding, or sensitivity to sample matrix, a thorough characterization of these factors and validation of the testing method would be required.

Finally, detection of endotoxins has also been reported using miniaturized chromatography methods. Makszin et al. used microchip electrophoresis (MCE) to separate and detect S-and R-type endotoxin components conjugated with fluorescence dyes. Separation took ~1 min and the limits of detection were 2.6 and 6.9 ng for the S- and R-type endotoxins [61]. Despite sensitive quantitation, the method required significant off-chip processing to perform the conjugation step.

To summarize, there already exist miniaturized methods validated for performing endotoxin testing of radiopharmaceutical formulations. Many other approaches have also been developed for endotoxin testing in other applications, which may offer advantages of speed, reagent cost, or convenience of readout if applied to radiopharmaceutical samples.

### 2.5. Chemical Purity and Identity

Chemical purity refers to absence of non-radioactive impurities in the formulated PET or SPECT tracer, including side products as well as residues of other components used in the production process. The purpose of testing is to ensure that the purification process has reduced residual amounts of impurities to safe levels (i.e., below allowed limits). The required chemical purity tests for radiopharmaceuticals depends on particular synthesis method and only needs to assess reagents added and byproducts expected for the particular synthesis route and conditions.

Though, in rare cases, a specific impurity can be determined via a simplified test (e.g., see Kryptofix test, Section 2.6), impurities are typically determined by chromatographic techniques, i.e., by performing chemical separation prior to detection. For some SPECT and PET radiopharmaceuticals that are labeled by a chelation reaction, the most significant impurity is unlabeled radioisotope, which can be detected via radio-TLC to determine (see Section 2.10). In other cases, impurities are typically quantified using high performance liquid chromatography (HPLC) combined with ultraviolet (UV) absorbance detection (HPLC/UV) or sometimes other modalities such as pulsed amperometric detection. Recently, analysis of radio-pharmaceuticals has also been performed using ultra-high performance liquid chromatography (UHPLC or UPLC), offering the advantages of faster separation times and more compact separation columns [62,63]. The identity of each peak in the detected chromatogram is determined by comparison of retention time with reference standards, and the quantity is generally determined from the peak area. For known impurities, the amount present in the sample is compared with allowed regulatory limits. For unknown impurities, so long as the impurities are below the limit of observed adverse effects in preclinical toxicology studies, they may be safe for injection. More specifically, it is not required to identify all impurities if microdosing criteria are met, i.e., their total mass is <100 μg and if the injected dose contains <1% of the pharmacologically active dose (determined using the same formulation as used in preclinical studies).

**Figure 5:**
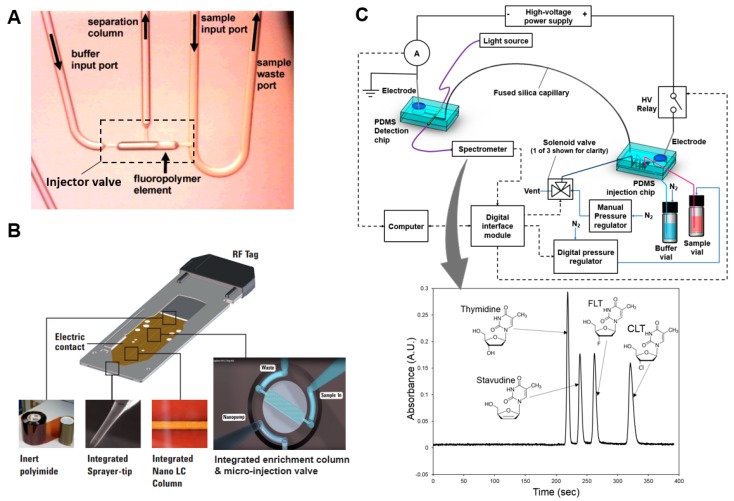
Examples of miniature chromatography systems. (**A**) Microfluidic HPLC system. A sliding fluoropolymer element is used to create a high-pressure valve for sample injection. When the element is in the left position, sample can be loaded via the sample input port. When the HPLC pump is activated, the flowing buffer closes the valve (slides the element to the right) and sweeps the injected sample through the integrated monolith column for separation. Adapted with permission from [64] Copyright © 2005 American Chemical Society. (**B**) Commercial HPLC-chip (Agilent)) includes an integrated micro-rotary injection valve with sample enrichment column serving as the injection loop, for injecting samples into the integrated LC column and downstream electrospray emitter to nebulize and transfer the sample to a mass spectrometric detector. Adapted from [65]. (**C**) Microchip electrophoresis setup with a volumetric sample injection chip, a separation capillary, and an optical detection chip. Inset shows representative electropherogram showing baseline separation of a mixture of 4 compounds. Adapted with permission from [74] Copyright © 2017 Elsevier.

Miniaturization of HPLC is one approach that could potentially be used for microscale implementation of the chemical purity test, though it has not yet been demonstrated for radiopharmaceuticals. In “microchip HPLC”, the HPLC column, and often elements of the injection valve, are integrated into a microfluidic chip. One implementation uses trapped cylindrical plugs of polymer monolith (formed by in situ polymerization) that slide within cylindrical glass microchannels to inject samples into the on-chip polymer monolith column [64] (Figure 5A). Another implementation that has been commercialized (HPLC-chip, Agilent, Santa Clarita, CA, USA) includes an integrated micro-rotary injection valve with sample enrichment column serving as the injection loop. In this setup samples are injected into an integrated column, flowing to a downstream electrospray emitter to nebulize and transfer the sample to a mass spectrometric detector [65] (Figure 5B). A recent review discusses the wide range of available microchip HPLC systems [66]. With chip-based HPLC, sample volume and separation time are significantly reduced (usually nL to µL), as is the physical size of the separation medium. However, chip-based HPLC systems still rely on bulky instruments that house the high-pressure pumping system, injection valve actuator, and detection modules, though efforts are underway to shrink these other components as well [66,67].

Capillary electrophoresis (CE) is another separation method in which a sample is driven through a separation medium by application of an electric field. Species are separated based on electrophoretic mobility in the separation buffer, and sometimes based on additional interactions with functionalized particles within the capillary (capillary electrochromatography, CEC) or by partitioning of analytes between micelles and surrounding buffer (micellar electrokinetic chromatography, MEKC). Unlike HPLC, CE can readily be miniaturized into microfluidic chips that are simple to fabricate and operate without the need for high-pressure pumps [68,69,70]. Additionally, CE exhibits high separation resolution and can achieve high detection sensitivity, and has been employed in diverse applications such as protein separation and pharmaceutical analysis [71,72,73]. By replacing conventional HPLC methods with microchip electrophoresis (MCE), there is much potential to significantly reduce the size, cost, and complexity of chemical purity testing systems in the future. Furthermore, sample volume is generally reduced by orders of magnitude, and very short analysis times (seconds) have been reported [68,74,75].

While there are numerous examples of MCE employed for pharmaceutical analysis [73,76], there have been very few examples of CE methods, and no examples of MCE methods, applied to the analysis of radiopharmaceuticals. Separation and detection of ^99m^Tc-labeled SPECT compounds has been performed with capillary zone electrophoresis and isotachophoresis [77,78,79,80,81], and use of CE has been suggested for analysis of PET tracers in a patent application [30]. Recently, using the PET tracers [^18^F]FLT and [^18^F]FAC as model systems, our group explored the feasibility of using micellar electrokinetic chromatography (MEKC) to separate neutral tracers from neutral impurities, and showed comparable separation and limit of detection as HPLC/UV [82]. We further showed that the system could be implemented as a hybrid MCE device (unpublished work), and that baseline separation of FLT from its known impurities and very repeatable injections could be achieved using only 4 nL of sample (Figure 5C) [74].

While our work has focused on detection via UV absorbance as a proof of concept, additional modes of detection can be implemented using techniques reported in the literature for pulsed amperometric detection [83], capacitively coupled contactless conductivity detection (C4D) [84,85,86], refractive index detection [87], or mass spectrometry [88,89]. For analysis of certain radiopharmaceuticals or impurities (e.g., with low UV absorbance), such alternative detection modes may be essential.

Efforts to miniaturize chemical purity testing for radiopharmaceuticals are just beginning, but chip-HPLC or MCE could provide a means to analyze a wide variety of PET and SPECT tracers with high sensitivity and separation resolution.

### 2.6. Kryptofix 2.2.2 (K222)

2,2,2-Cryptand or 4,7,13,16,21,24-hexaoxa-1,10-diazabicyclo [8.8.8]hexacosane, also known by its commercial name Kryptofix 2.2.2 (K222), is frequently used as a phase transfer catalyst in the manufacture of ^18^F-labeled PET tracers. Radiopharmaceutical formulations must be analyzed for residual amounts of K222 before human application due to its toxicity. The limit specified in the US Pharmacopoeia (USP) is <50 µg/mL and EP limit is 2.2 mg/V (i.e., per patient). Most commonly, residual K222 is assessed qualitatively via a TLC method in which the size and intensity of spot of K222 from the sample solution should not exceed (by visual observation) that of a spotted reference solution [90]. Another method uses a spot test in conjunction with iodoplatinate indicator, and can be completed in 5 min (compared to 30 min for the TLC test) [91]. As with any colorimetric test, there is some risk of non-specific interactions that could interfere with the test result. For example, the spot test is sensitive to tertiary amines, which may be present in the radiotracer itself or impurities, potentially leading to false positives. To avoid such issues, K222 is sometimes analyzed after chromatographic separation. Reports have shown that K222 can be detected via gas chromatography (GC) with a nitrogen detector [92], liquid chromatography with mass spectrometric (MS) detector (LC/MS [93,94] or UPLC/MS [95]) or HPLC with a UV detector (HPLC/UV) with post-column and pre-column derivatization [96].

Efforts to miniaturize indicator-based tests for radiopharmaceuticals in microfluidic chips have been reported. For example, the group at the University of Hull reported the on-chip optical absorbance-based detection of K222 by first mixing the sample with iodoplatinate reagent then loading the sample into a microchannel [97,98]. The later report indicates a limit of detection of 28 ppm. Presumably this approach could be combined with an on-chip mixer, and could leverage approaches for high-sensitivity optical detection systems (see Section 2.1) to further improve sensitivity.

Other colorimetric K222 indicator chemistries can likely be used in microfluidic devices as well. Anzellotti et al. reported an evaluation of the I_2_/I^−^ indicator for testing PET radiopharmaceuticals and suggested that it could be used to test sample volumes as low as 2–25 µL [99]. Experiments showed that the color change occurred within 1–2 s, and that the indicator was unaffected by the presence of several salts or amine-containing PET tracers in the formulation.

Alternatively, the K222 test could potentially be implemented in miniaturized form using microscale chromatographic approaches. For example, we have shown, using capillary electrophoresis, that K222 could be separated from FLT and several impurities and identified and quantified in <2 min [82], though miniaturized detection in an MCE device has not yet been demonstrated.

Although initial efforts have been made to miniaturize the K222 test for radiopharmaceuticals, further development is needed to demonstrate reliability and ease of use for implementation in a radiopharmaceutical QC facility.

### 2.7. Residual Organic Solvents

Residual solvents in the final formulation could exhibit a variety of toxic effects and the remaining amounts of all solvents used during the synthesis and purification process must be monitored to ensure residual amounts are below safe limits. Typically this test is needed for PET tracers, but often can be omitted for SPECT tracers when produced under aqueous reaction conditions. There is clear guidance on the limits for many possible solvents [100]. For the commonly used class 2 solvents acetonitrile (MeCN), dichloromethane (DCM), and *N*,*N*-dimethylformamide (DMF), the allowed limits are 4.1 mg/day (410 ppm), 6 mg/day (600 ppm) and 8.8 mg/day (880 ppm), respectively. Class 3 solvents such as ethanol (EtOH) and dimethylsulfoxide (DMSO) have much lower toxicity, and up to 50 mg/day (5000 ppm, 50 mg/V) is allowed for each. Typically, residual solvents are assessed using gas chromatography (GC) in conjunction with a flame ionization detector. Testing can also be performed via HPLC in conjunction with a refractive index detector [21,101].

For the particular application of radiopharmaceutical analysis, a recent preliminary study suggests that residual solvent impurities might be quantified via raman spectroscopy in a glass microfluidic flow cell [102], though details of the method and its performance have not yet been published. A patent application also mentions the possibility to detect solvents in radiopharmaceuticals via a gas sensor microarray (“electronic nose”) [103] microelectromechanical systems (MEMS) device, though details are not described [104]. While, in principle, it seems possible that residual solvents could also be assessed via chip-HPLC systems [66,67] (e.g., with RI detection) or microscale GC systems [105,106], these approaches have not yet been demonstrated.

Further development is therefore needed to implement a reliable microfluidic detection method for organic solvents with the required performance for testing of radiopharmaceuticals.

### 2.8. Radioactivity Measurement (Radioactivity Concentration)

The concentration of radioactivity in every batch of the final drug preparation at the end of synthesis (EOS) must be measured [10]. This value is needed to determine how much volume to dispense for each individual patient dose (decay-corrected to the expected time of injection). The total amount of radioactivity in the vial containing the final drug preparation is usually measured using a calibrated dose calibrator. In order to determine the concentration, a known volume of the final drug preparation is withdrawn into a syringe, the activity of the syringe is measured, and the concentration then calculated.

**Figure 6:**
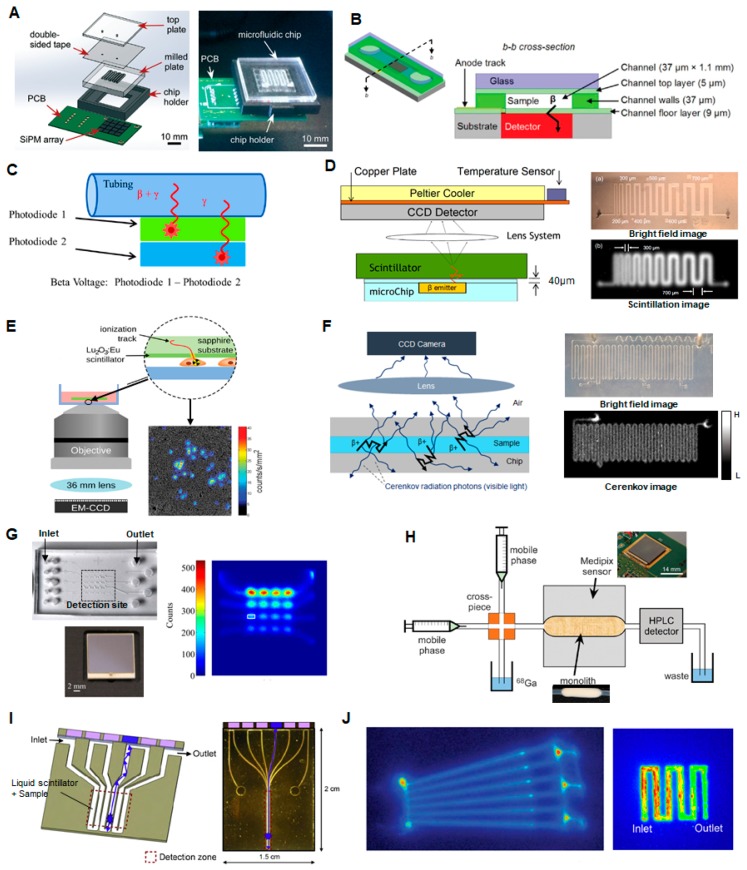
Examples of radiation detection methods (**A**) (**left**) Setup of microfluidic chip and silicon photomultiplier (SiPM) array for measurement of radioactivity and half-life of a sample. (**right**) Photograph of the final chip setup on the SiPM array. Adapted from [107] with permission of the Royal Society of Chemistry. (**B**) Device for positron detection from blood samples in a microchannel bonded to a silicon PIN photodiode. Adapted from [108] with permission of the Royal Society of Chemistry. (**C**) Dual silicon PIN photodiode detection of a sample in a channel or tubing. Photodiode 1 responds to both positrons and gamma rays, while photodiode 2 responds only to gamma radiation, allowing subtraction of the background gamma signal. Adapted with permission from [109]. Copyright © 2016 Springer. (**D**) Scintillator based detection. A scintillator was placed in close proximity to a PDMS microfluidic chip and scintillation light from positron interactions was detected with a CCD camera. Adapted with permission from [110] Copyright © 2007 IEEE. (**E**) Radioluminescence imaging. Scintillator light output is observed via sensitive microscope as radioactive decays occur in sample. Adapted with permission from [111] Copyright © 2015 John Wiley & Sons, Inc. (**F**) Cerenkov imaging. (**left**) Cerenkov radiation is emitted as energetic particles travel through the liquid or chip material. (**right**) Bright-field and Cerenkov images of 200 µm-wide microchannel filled with [^18^F]FDG solution. Adapted from [112] with permission of IOP PUBLISHING, Ltd. (**G**) Beta-box imaging. (**top left**) Microfluidic chip containing multiple sample chambers is placed on the detector; (**bottom left**) photograph of position sensitive avalanche photodiode (PSAPD) detector; (**right**) resulting image when sample chambers are filled with varying concentrations of [^18^F]fluoride. Adapted from [113] with permission of IOP PUBLISHING, Ltd. (**H**) System for measuring radioactivity in a silica-based monolith column placed above a Medipix sensor. Adapted from [114] with permission of the Royal Society of Chemistry. (**I**) Microfluidic channel filled with liquid scintillator that acts an array of scintillation detectors connected by liquid waveguides to photodetectors. Adapted with permission from [115] Copyright © 2010 Elsevier. (**J**) Autoradiography (phosphor imaging system). (**left**) Autoradiography image of adsorbed radiolabeled peptide on the channel surfaces of a plastic microfluidic chip. Adapted from [116] with permission of The Royal Society of Chemistry. (**right**) Autoradiography image of [^18^F]fluoride trapped in the serpentine channel of an electrochemical cell. Adapted with permission from [117] Copyright © 2013 Elsevier.

To miniaturize this test, the sample can be loaded into a microfluidic channel placed in close proximity to a compact radiation detector. Taggart et al. have shown that a small array of silicon photomultipliers (SiPMs) could measure the radioactivity in a sample of PET or SPECT radiotracers ([^18^F]FDG, [^68^Ga]gallium citrate, [^99m^Tc]pertechnetate) contained in an adjacent 40 µL serpentine microchannel after stopping the flow (Figure 6A) [107]. The effect of different microfluidic chip materials and substrate thicknesses were investigated. In their optimized device, good linearity was observed for the PET tracers over a range of ~0.01 to 100 MBq total activity, though upper and lower limits were not reported. The signal for the SPECT tracer required longer integration time to obtain a reliable reading. Convert et al. have demonstrated a miniaturized device for a slightly different application: Measuring the radioactivity concentration in the blood of a rodent model for pharmacokinetic analysis of PET and SPECT radiotracers [108]. To maximize detection efficiency, a polymeric microfluidic device with a 0.7 µL microchannel was bonded directly on top of a commercial unpackaged 300 µm thick silicon PIN photodiode, with only 9 µm separation between the sample and detector (Figure 6B). Limits of detection were not reported, but absolute detection efficiency of the microfluidic geometry was significantly higher compared to a capillary-based setup, and detection efficiencies were close to the theoretical maximum for ^11^C- and ^18^F-decay events (47% and 39%, respectively, compared to 50% theoretical maximum), due to the high efficiency of direct positron detection. Dooraghi et al. reported radiation measurement of [^18^F]fluoride and [^18^F]FDG solutions in capillary tubing or a microchannel to calibrate an automated aliquoting / dose-dispensing system [109]. The detector comprised two 3 mm × 30 mm silicon PIN diodes at different distances from the sample such that the closest detector was sensitive primarily to positron interactions, while the further detector allowed measurement and subtraction of background gamma interactions (Figure 6C). The dynamic range of detectable activity was measured to be 0.74–4400 kBq/µL (0.02–120 µCi/µL) and 0.01–105 MBq/µL (0.3–2830 µCi/µL) for the high- and low-gain electronics configurations, respectively.

Additional compact radiation detection methods have been reported for applications that require a higher degree of spatial information about the radioactivity distribution of positron-emitting samples, but presumably these could be adapted to measure the radioactivity concentration in sample of a formulated PET radiopharmaceutical.

Cho et al. reported a system in which a scintillator was placed in close proximity to a PDMS microfluidic chip and scintillation light from positron interactions was detected with a CCD camera [110,118] (Figure 6D) Preliminary studies showed that this imaging device is capable of discerning line pairs of printed [^18^F]FDG solution with separation as low as 300–500 µm and quantifying the activity concentration reliably down to 1.48 Bq/mm^2^ (40 pCi/mm^2^) or 13.32 Bq/mm^2^ (360 pCi/mm^2^) in 5 min with the use of CsI or plastic scintillators, respectively. Pratx et al. reported a different scintillation-based approach known as radioluminescence microscopy [119] in which the sample was placed on a thin scintillator plate, and light produced by positron decay events was measured using a high-sensitivity microscope (Figure 6E). The spatial resolution, measured using a dried droplet of [^18^F]FDG (370 MBq), was estimated to be 5 µm FWHM, and the minimum detectable activity density was 150 Bq/mm^2^ (4 nCi/mm^2^). The high sensitivity allowed detection of [^18^F]FDG uptake in single cells. The maximum detectable activity was not specified.

Cho et al. demonstrated an additional measurement technique in collaboration with our group: a sensitive CCD camera was used to detect the Cerenkov radiation emitted as energetic positrons from the sample travel through the surrounding medium [112,120] (Figure 6F). Using solutions of [^18^F]fluoride and [^18^F]FDG, the system dynamic range was found to be 7.03–2740 kBq/mm^2^ (0.19–742 μCi/mm^2^) for 5 min acquisitions, though the upper end of the dynamic range could be further improved by using a shorter camera acquisition time and a smaller lens aperture setting, if necessary. The spatial resolution of Cerenkov image was found to be ~200 µm.

Dooraghi et al. developed a system based on direct positron detection using a position-sensitive avalanche photodiode (PSAPD) (Figure 6G) [113]. In a PSAPD, the interaction of a positron creates an electron-hole pair that is amplified by an avalanche affect due to an applied bias across the device, allowing positron interactions to be easily distinguished from thermal noise. Furthermore, a 4-point detector allows localization of the detection event across the substrate. The sample is contained in a chamber on a PDMS microfluidic chip with 50 µm thick PDMS substrate separating the fluid from the detector. For ^18^F-containing solutions, the lower detection limit was measured to be 0.5 Bq/mm^2^ (~14 pCi/mm^2^) for a 1 min acquisition and 62 × 62 μm pixel size. The same device has been used to quantify activity in single cells [121]. The upper count rate limit was 21,000 cps (~46 kBq). The spatial resolution ranged from 0.4 mm FWHM at the center of the field of view (FOV), and 1 mm at a distance of 5 mm away from center.

Tarn et al. have demonstrated the use of the commercially available miniaturized positron sensor array (Medipix2, Medipix Collaboration) with a pixel size of 55 µm × 55 µm and overall detector size of 14 mm × 14 mm (65 k pixels) to measure activity of [^68^Ga]Ga-citrate solutions in Tygon tubing (709 nL detection volume, 254 µm sample to detector distance). Upper and lower detection limits were not reported but a linear response was observed over the range tested (0.05–100 MBq/mL). The detector was also used to measure the activity passing through a silica monolith (Figure 6H) [114] as described in Section 2.10. Maneuski et al. reported the use of a similar detector (Timepix) for measurement of activity in an adjacent tubing designed to measure the output of a UPLC system [122]. Though complete details of geometry were not provided, if the tubing is assumed to have a standard 1.6 mm (1/16″) outer diameter, then the distance from sample to detector is ~400 µm. Injected samples with concentration as low as 1 kBq/mL could be detected. Sensitivity was found to be much greater than several scintillation-based detectors.

Mapelli et al. have reported an interesting detector comprising a serpentine microfluidic channel filled with liquid scintillation material [115,123] (Figure 6I). Each path of the serpentine channel acts as an independent detector to convert radiation into light in one portion of the channel (via the liquid scintillator), and also as a liquid waveguide to guide this light to a distant photodetector in a non-irradiated portion of the device. The idea was to provide improved detector resolution and increased radiation resistance, while achieving efficient optical coupling throughout the system and potentially enable fabrication of low-cost, high-resolution detectors. The photoelectric yield of the chip was measured by exciting the liquid scintillator with electrons from an external collimated ^90^Sr source and found to be in the order of 1.65 photoelectrons per minimum ionizing particles (MIP) for 200 µm deep microchannels. Presumably this device could be used to measure the activity of radiopharmaceutical samples loaded into an adjacent microfluidic chip.

Another technique that has been used to analyze compounds labeled with C-11, Ga-68, or F-18 in microfluidic channels is autoradiography (Figure 6J) [116,117]. Laven et al. did not report the detection limit but reported good linearity in the range of ~10–180 Bq. The need to first expose then image the phosphorimaging plates could make this approach too slow and impractical for an automated QC testing system, and too bulky for microscale implementation.

The examples described here represent a wide variety of detection approaches that may be suitable for measuring radioactivity in microchannels. With further development, it is likely that several of these approaches could provide a compact means to perform radioactivity measurements for the radioactivity test (as well as some of the tests below).

### 2.9. Radionuclidic Purity and Identity

The radionuclidic purity is the ratio of the desired radionuclide activity to the total activity. The purpose of testing is to ensure that the radiopharmaceutical is not contaminated with other radionuclides, that could make the resulting PET/SPECT images difficult to interpret, or, in the case of long-lived isotopes, could cause harm to the patient. For example, SPECT and PET tracers labeled with generator-produced radionuclides can contain contamination by radioactive parent compounds. In general, the radionuclidic identity can be confirmed by measuring the half-life of the product, and the radionuclidic purity can be measured by a gamma spectrometer with a multichannel analyzer (e.g., based on sodium iodide scintillation detector or high-purity germanium detector). The half-life is determined by taking at least 3 radioactivity measurements of a sample using a dose calibrator (with the same geometry for each measurement), and then calculating half-life via a nonlinear fit to the radioactivity decay equation [17]. For highest accuracy, the measurements should be carried out as far apart in time as practical. The radionuclidic purity is determined by obtaining a gamma spectrum of the sample, and matching this spectrum (after background spectrum is subtracted) to the expected energy spectrum. Each radionuclide has a characteristic gamma spectrum; for example, positron-emitting isotopes in PET tracers have a peak at 0.511 MeV due to positron annihilation, plus additional peaks corresponding to other decay modes, if applicable. Since some positron emitters have a very short half-life, the USP and EP allow PET radiopharmaceuticals to be released before the radionuclidic identity test is completed on each batch, in which case periodic tests of samples prepared in the same way would be necessary.

The half-life test can be miniaturized using an integrated radiation detector to measure the radioactivity of a sample in a microchannel in a consistent geometry multiple times. Taggart et al. used their SiPM array platform to measure not only the radioactivity concentration as described above but also the half-life of microfluidic samples [107]. Accurate half-life measurements were achieved for [^18^F]fluoride and [^18^F]FDG solutions based on 5 min measurements taken over a time period of several hours, but reliable values could not be obtained for calculations based on shorter measurements (e.g., 30–60 s) taken over a more realistic 20 min timespan without constraining the exponential fit. It is possible that performance could be improved with further optimization. In addition, any of the microscale radiation detection approaches described in Section 2.8, depending on activity level and geometry of the sample, could be considered for microscale implementation of the half-life test.

To the best of our knowledge, a microscale implementation of the radiouclidic purity test has not yet been demonstrated, though perhaps a microscale gamma spectrometer could be implemented using similar technologies to those discussed in Section 2.8, provided the radiation detector has adequate energy resolution.

### 2.10. Radiochemical Purity and Identity

The radiochemical identity of a positron-emitting radiopharmaceutical is generally determined after chromatographic separation by determining the retention time (radio-HPLC), retardation factor (radio-TLC), or migration time (radio-CE) of the main radioactivity peak (corresponding to the radioactive product), and comparing to that of a reference standard to confirm structural identity of the radiopharmaceutical.

In addition to confirming the identity of the product, it is also necessary to ensure the absence of radioactive impurities. Radiochemical purity is defined as the ratio of the activity of the radionuclide concerned, which is present in the desired chemical form, to the total activity of that radionuclide present in the radiopharmaceutical preparation. Any radiochemical impurity can potentially affect the biodistribution of the radiopharmaceutical giving a misleading imaging result. In general, the radiochemical purity should be >95%, though in the case of [^18^F]FDG, USP specifies >90% as acceptable.

Typically, radiochemical identity and purity are determined via HPLC with a radiation detector positioned at the end of the column (radio-HPLC). While the same instrumentation can be used for the chemical purity test, the radiochemical identity and purity tests are often run on a sample that is spiked with a non-radioactive reference standard, while the chemical purity test is carried out on an unadulterated sample. The standard is used to confirm the radiochemical identity by matching retention times of the radiation and UV absorbance peaks in the chromatogram. In addition to radio-HPLC, analysis by radio-TLC may also be necessary for accurate determination of radiochemical purity since certain radioactive species such as [^18^F]fluoride can be under-represented in HPLC [124]. In the case of some PET and SPECT tracers labeled via chelation reactions, where only the product and non-chelated radionuclide are expected in the final formulation, radio-TLC may be sufficient (without radio-HPLC) for determination of purity.

Similar to the corresponding non-radioactive tests, there is interest in developing miniaturized versions of the radiochemical identity and purity tests due to the bulkiness of radio-HPLC, radio-TLC, and radio-CE systems. As discussed in Section 2.5, HPLC and CE can be miniaturized into chip-HPLC or MCE format, and potentially these techniques could be integrated with one of the compact radiation detection approaches described in Section 2.8 to measure the output of the separation column or separation channel, respectively. To the best of our knowledge, such integration has not yet been reported.

Some efforts have been made to replace traditional HPLC/UPLC radiation detectors with more compact detectors, which could be a step in this direction. For example, the Timepix detector reported by Maneuski et al. [122] was suitable for measurements at the output of a UPLC system and showed a wider dynamic range and improved sensitivity compared to a photomultiplier tube (PMT) detector with either CsF or BGO scintillator.

Tarn et al. have used a Medipix detector to detect radioactivity within a porous silica monolith embedded in tubing with the goal of real-time imaging of separation processes. The detected signal was higher for a sample of [^68^Ga]Ga-citrate passing through the monolith than the signal observed with a traditional radio-HPLC detector (NaI scintillator/PMT) positioned downstream in the tubing. In addition, the accumulation of activity could be measured as [^68^Ga]Ga^3+^ was trapped on the monolith, as could the reduction in activity when the monolith was eluted. Maneuski et al. also demonstrated the spatial imaging capabilities of the Timepix detector in the context of radiochemical separations. The radioactivity distribution along a conventional radio-TLC plate containing an unspecified ^18^F-labeled sample was measured with sub-mm resolution by placing it adjacent the detector [122]. Imaging of three parallel ^18^F-solution-containing tubes was also demonstrated, perhaps in anticipation of performing chromatographic separations in channels in the future. Spatially-resolved radiation detection was also mentioned in a patent by Hansteen et al. [30] to monitor separation of radiopharmaceuticals in a capillary to determine radiochemical purity, though details were not provided.

While some steps have been taken toward miniaturized implementation of radiochemical identity and purity tests, much development is still needed in this regard.

### 2.11. Molar Activity

Molar activity (often called “specific activity”) is a measure of the amount of radioactivity per molar amount of the radiopharmaceutical [3]. For certain radiopharmaceuticals, such as [^18^F]FDG, radiolabeled amino acids, or fatty acids that visualize metabolic processes, molar activity determination is not crucial since these radiotracers have natural physiologically abundant levels of nonradioactive counterparts in vivo, especially in circulation. However, for receptor-binding radiotracers, radiolabeled mAbs, and peptide hormone analogs, a relatively high molar activity is crucial. Conventionally, for most organic radiopharmaceuticals, the concentration (total number of moles per volume) is determined based on analytical HPLC after creating a calibration curve using known concentrations of an ultra-pure reference standard. If radioactivity concentration is also known, then molar activity can easily be calculated.

Though it has not been demonstrated, molar activity measurement can likely be miniaturized by combining aforementioned techniques for measuring concentration (chemical purity test, Section 2.5) and radioactivity concentration (radioactivity test, Section 2.8). High-sensitivity and accuracy will be needed to measure the concentration, as the molar amounts can be very small (e.g., pmol to nmol for ^18^F-labeled PET tracers).

## 3. Outlook

In this paper, we have reviewed various microfluidic implementations of the QC tests for PET and SPECT radiopharmaceuticals, or techniques that could potentially be implemented to perform these tests (summarized in Table 1). It is encouraging that viable approaches for ultra-compact implementation of many of the required tests have been demonstrated at least as a proof of concept. In some cases, the desired performance has already been demonstrated, and/or significant reductions in sample consumption have been reported.

However, in most cases, considerable development and optimization efforts will be needed to increase reliability, speed, sensitivity or other measures of performance up to the rigorous requirements of clinical testing. In addition, in cases where the method of detection is fundamentally changed (e.g., sterility testing based on counting of individual bacterial, assessment of chemical/radiochemical purity via new chromatographic approaches such as MCE, and detection of residual solvents via Raman spectroscopy or an “electronic nose”), extra efforts may be required to demonstrate equivalence (or superiority) to currently-accepted test methods.

Once individual tests have been perfected, efforts will be needed to integrate them into a unified system that performs all tests, data collection, and report generation in a fully-automated manner. Integrated systems with some degree of microfluidic elements have been suggested in patents and patent applications [30,104] and detailed implementations have recently been described [98,125]. One of the challenges is in forming fluidic interconnections among all devices and delivering aliquots of the sample to each one. The possibility to integrate sample distribution channels, sample channels/chambers and detectors into a single lab-on-a-chip device could minimize the overall fluidic complexity and potential failure points. Another challenge is in avoiding cross-contamination from one sample to the next, which may require extensive cleaning protocols and cleaning validation. However, because microfluidic elements can often be fabricated using very low-cost materials and methods, it may be possible to implement the tests using disposable fluid paths. Leveraging such features could eliminate the need for cleaning, and could reduce the required maintenance, further simplifying the testing process and overall QC testing platform.

If realized, integration of microscale QC tests on an automated platform would allow for unified QC system validation, eliminate operator-induced variation, significantly reduce radiation exposure to personnel, and streamline the overall workflow. As increasing numbers of the thousands of known tracers [126,127] move into the clinic, and as new technologies such as microfluidic systems enable more widespread production of tracers on demand [24,128,129,130], it will become increasingly important to have an integrate QC testing platform that simplifies and reduces the cost of QC testing.

Though we have focused on the special needs of radiopharmaceutical analysis, where sample volume and total duration are extremely limited, the methods described here could likely also be applied to the analysis of non-radioactive pharmaceuticals, and may offer significant advantages in terms of speed and cost.

## Figures and Tables

**Table 1:** Summary of required QC tests, the conventional method(s) used, and typical specifications. In addition, examples of microfluidic approaches to perform each test are listed. Note we have indicated in italics approaches that have not been demonstrated/proven, but may be possible in principle. Abbreviations: HPLC/UV = HPLC with UV absorbance detector, HPLC/RI = HPLC with refractive index detector, etc.

QC Test	Conventional Method (s)	Typical Acceptance Criteria	Examples of Microfluidic Suitable Approaches
pH	pH indicator strips; electronic pH meter	4.5 < pH < 8.5	Absorption spectroscopy of sample + indicator [31,34,35] Absorption spectroscopy of pH-sensitive surface [37] Fluorescence emission from pH-sensitive dye/surface [36] Hydrogel-based pH sensing (physical or electrical change) [38,39,40] Electrochemical cell [41,42]
Appearance (color/clarity)	Visual	Clear, colorless, particulate-free	Absorption spectroscopy of sample [31]
Sterility	Short term: filter integrity test (e.g., bubble point test); long term: Bacterial culture	Long term: No bacterial growth observed	Fluorescent detection of “universal” dye that binds to bacteria [50] Electrical impedance detection of individual bacteria [51,52,53]
Bacterial endotoxin	LAL test	175 EU/V	Variations of LAL assay (absorption spectroscopy detection [56,57], electrical impedance detection [58], bioluminescence detection [59]) Detection of endotoxin binding to surface [55,60] MCE/fluorescence [61]
Chemical identity/purity	HPLC/UV	Varies	MCE/UV [74] Chip-HPLC/UV
Kryptofix 2.2.2	Color spot test	<50 µg/mL (USP); 2.2 mg/V (EP)	Absorption spectroscopy of sample + indicator [97,99] MCE/UV Chip-HPLC/UV
Residual organic solvents	Gas chromatography; HPLC/RI	Varies (e.g., MeCN 4.1 mg/day, EtOH 50 mg/day, DMSO 50 mg/day, DCM 6 mg/day, DMF8.8 mg/day)	MCE/RI Micro-GC *Electronic nose* [103]
Radioactivity concentration	Dose calibrator	Varies	Solid-state detectors (SiPM [107], PIN diode [108,109], PSAPD-based detector [113,131], Medipix/Timepix detector [114,122]) Cerenkov imaging [112,120] Scintillator-based detectors (CCD imaging [110,118], radioluminescence microscopy [111,119], liquid scintillator with photodetector [115])
Radionuclidic identity	Half-life measurement with dose calibrator	Varies (e.g., 105-115 min for ^18^F-labled tracers)	Radiation detector (half-life measurement) [107] Potentially some of radiation detectors listed under “Radioactivity concentration” can be used
Radionuclidic purity	Gamma spectrometer	Match expected energy spectrum	Potentially some of radiation detectors listed under “Radioactivity concentration” can be used
Radiochemical identity and purity	Radio-HPLC; radio-TLC	>95%; (>90% for [^18^F]FDG)	Porous silica monolith with Medipix positron detector array [114] TLC plate with Timepix positron detector array [122] MCE/positron detector Chip-HPLC/positron detector
Specific activity	Radio-HPLC and dose calibrator	Varies	MCE/UV and radioactivity measurement Chip-HPLC/UV and radioactivity measurement
